# The importance of the optic nerves unlocking during the resection of anterior skull base meningiomas for visual function preservation: surgical nuances and clinical outcome

**DOI:** 10.1007/s10143-025-03210-z

**Published:** 2025-01-09

**Authors:** Carmelo Lucio Sturiale, Carolina Noya, Gianluca Trevisi, Rina Di Bonaventura, Lara Brunasso, Rosario Maugeri, Alessandro Olivi, Alessio Albanese

**Affiliations:** 1 Department of Neurosurgery, Fondazione Policlinico Universitario A. Gemelli IRCCS, Università Cattolica del Sacro Cuore, L.go A. Gemelli 8, 00168 Rome, Italy; 2 Department of Neurosciences, Imaging and Clinical Sciences, G. D’Annunzio University, Chieti‑Pescara, Italy; 3 Neurosurgical Clinic, AOUP “Paolo Giaccone”, Post Graduate Residency Program in Neurologic Surgery, Department of Biomedicine Neurosciences and Advanced Diagnostics, School of Medicine, University of Palermo, 90127 Palermo, Italy; 4 Institute of Neurosurgery, Università Cattolica del Sacro Cuore, L.go A. Gemelli 8, 00168 Rome, Italy

**Keywords:** Optic nerve, Meningioma, Chiasm, Unlocking strategy, Visual acuity, Visual field, Falciform ligament, Clinoidectomy, Unroofing

## Abstract

Anterior skull base meningiomas can determine optic nerves (ONs) impingement leading visual disturbances as presenting symptoms. According to the relationship between tumour origin and ON course, different “vectors of compression” can be identified: lateral-to-medial, medial-to-lateral, inferior-to-superior, and anterior-to-posterior. As visual function preservation represents the main goal of surgery, we designed a procedural algorithm concerning approach, cisterns dissection, falciform ligament section, ON mobilization and tumour debulking aimed to reduce ONs manipulation during surgery. We included 40 patients harbouring meningiomas compressing ONs with mean age 61.7 ± 12.4 years. Sixteen originated from anterior clinoidal process (40%), 10 from sphenoid-ethmoidal planum (27.5%), 10 from tuberculum sellae (25%), and 4 from sphenoid-orbital region (7.5%). A decline in visual acuity was observed in 34/40 (85%) of patients and in visual field in 28/40 (70%). Mean age appeared significantly lower in patients with intact visual field (*p* = 0.006). No differences were observed between symptomatic and asymptomatic patients according to tumour origin, whereas a significantly lower rate of visual field impairment was observed among patients with inferior-to-superior compression. On the contrary, tumour determining a superior-to-inferior compression showed a trend of higher risk of visual field cut. Falciform ligament opening was performed in 82.5% of cases, optic canal unroofing in 27%, anterior clinoidectomy in 32% and optic strut removal in 5%. At 6-month follow-up, none among patients treated before of visual acuity onset disturbances showed worsening. Among those showing preoperative alterations, an improvement was observed in 17/34 (50%), 14 (41.2%) had an unchanged deficit, and 3 (8.8%) a worsening. Optic canal unroofing was the only significant predictor of postoperative non-improvement at multivariate analysis (*p* = 0.03, AUC = 0.796; OR = 0.163; 95%CI:0.027–0.983; *p* = 0.04). Similarly, none patient developed visual field cut when treated before it clinical appearance, and only 28.6% of those with a pre-operative deficit showed post-operative improvement. Worsening was seen in 5/28 of patients with a preoperative visual field deficit (17.6%), with the remaining 15 (53.6%) with unchanged visual field at 6-month. Comparing patients with post-operative visual field improvement and non-improvement, only a younger age and a better preoperative mRS status showed a significant association with a positive outcome. Age emerged as unique significant risk factor for lack of post-operative improvement at stepwise binomial logistic regression model (OR = 0.855, 95%CI: 0.743–0.983, *p* = 0.028). The surgical management of anterior cranial fossa meningiomas associated with optic nerve compression should prioritize visual preservation over radical tumor resection and a timely decompression reducing the risk of post-operative visual acuity deterioration. The surgical techniques should be also modified to include all the necessary unlocking strategies limiting the ON stress during the tumor manipulation.

## Introduction

Anterior cranial fossa meningiomas often pose significant surgical challenges due to their relationship with optic nerves (ONs) and chiasm, potentially leading to visual impairment at clinical onset [2, 5–8, 15].

Although they are slow-growing lesions, their proximity with the anterior visual pathway can lead to severe visual loss. These tumors tend to follow the path of least resistance, and as a result they can invade the entire the optic nerves, from the optic canal posteriorly to the orbit anteriorly. Treatment of this condition is controversial, due to the difficult to completely remove these tumors without causing secondary complications.

However, surgical resection can be justified in cases of unilateral extension to reverse visual deficits that have already occurred or to avoid extension to other areas and involvement of contralateral optic nerve or vital cerebrovascular structures.

The direction of tumor growth in relation to the ONs course plays a crucial role in surgical planning as it represents a measure of the possibility of transcranial tumor resection with optic pathways preservation [2, 5, 7]. In a previous study, we identified four main “vectors of compression”: lateral-to-medial, medial-to-lateral, inferior-to-superior, and anterior-to-posterior [7].

To safely remove these tumors and maximize the opportunity to preserve visual function, we developed a surgical algorithm that considers the specific vector of compression. This algorithm includes key-steps regarding microsurgical dissection of basal cisterns, opening of the falciform ligament (FL), mobilization of the ON, and tumor debulking [7, 12, 13].

A thorough understanding of the tumor’s location and its relationship with the ONs is essential for planning a successful surgical approach, since carefully considering these factors can minimize the risk of complications and improve visual outcomes.

The aim of this study was to evaluate the risk associated with optic nerve decompression during the tumor removal, and to measure the rate of visual deterioration after surgery performed according to the surgical principles of optic nerve unlocking that we had already reported in a previous study [7].

## Materials and methods

Based on the progressive technical refinements of our institutional experience of ON compression from extra-axial tumors, we analyzed the surgical steps necessary to release ONs and chiasm before the resection of anterior cranial fossa meningioma, which frequently involve their passive mobilization during the tumor debulking [7, 12, 13].

Then, we reviewed all the safety precautions that we adopted in different surgical settings and evaluated the clinical and visual outcome of patients, thus identifying fundamental key-points and designing an algorithm with different steps to obtain ONs unlocking. They include approach, microsurgical dissection of the basal cisterns, FL opening, unroofing of the ON canal, anterior clinoidectomy, optic strut removal, ON mobilization and tumor debulking.

Table 1 summarizes the algorithm applied for each tumor according to its origin and the direction of ON compression.

**Table 1:** Surgical steps according to the direction of the optic nerve compression

Direction of compression	Tumor origin	Bilateral optic pathway involvement	Falciform ligament section	optic canal unroofing	Anterior clinoid process drilling	Optic strut removal
Lateral to medial	Clinoid meningiomas	NO	YES	YES	YES	-
Medial to lateral	Diaphragm sellae meningiomas	YES	YES	YES	-	-
	Tuberculum sellae meningiomas	YES	YES	-	-	-
Inferior to superior (and medial to lateral)	Tuberculum sellae meningiomas	YES	YES	YES	-	-
Anterior to posterior	Olfactory groove meningiomas	YES	YES	-	-	-
Circumferential	Optic foramen meningiomas	NO	YES	YES	YES	YES

We included all patients affected of anterior skull base meningiomas associated with compression of the optic pathways that were surgically treated at Neurosurgical Unit of Fondazione Policlinico Universitario Agostino Gemelli IRCCS during the last 5 years (between 2019 and 2023). Recurrent tumors, pediatric patients, and patients treated via transnasal-transphenoidal endoscopic approach or affected by other tumors (for instance metastasis) were excluded from this study.

We retrospectively collected data about demographics, clinical and neuro-ophthalmological examinations (visual acuity test, visual field test, endocrinological evaluation, and mRS score at admission), radiological and histological tumor characteristics, surgical findings (approach, extent of resection, complications), clinical and visual outcomes.

The results of visual acuity test were stratified according to the 2010 WHO classification.

Visual eye field test and acuity test were performed by ophthalmologists with computerized methods.

Among global indices, the “mean deviation” (MD) and the “visual field index” (VFI) and their linear rates are commonly used.

Radiological investigations consisted in preoperative contrasted magnetic resonance imaging (MRI) of the brain and when available, a cerebral CT-scan with/without contrast.

Based on the preoperative images, the patients were divided in different subgroups according to:

- tumor origin: tuberculum sellae meningiomas, olfactory groove meningiomas, planum sphenoidalis, sphenorbital and anterior clinoidal process meningiomas extending either laterally to the carotid cistern or anteriorly to the planum sphenoidale and optic canal or posteriorly beyond the dorsum sellae;
- direction of optic pathways compression:

- lesions determining a lateral-to-medial compression, such as clinoid meningiomas;
- lesions determining a medial-to-lateral compression, such as tuberculum sellae meningiomas;
- lesions determining an inferior-to-superior compression (often in combination with a medial-to-lateral compression), such as tuberculum sellae meningiomas and diaphragm sellae meningioma;
- lesions determining an anterior-to-posterior compression, such as olfactory groove meningiomas and planum sphenoidal meningioma;

- presence or absence of laterality of the visual deficit: right, left, bilateral;
- tumour size, encasement of vascular structures and presence of calcifications.

Brain CT-scan was usually done on first postoperative day to evaluate postoperative complications, whereas a brain MRI was suggested within 3 months from surgery for residual tumor evaluation. All patients were assessed by an ophthalmologist before and after surgery and all patients with visual deficits continued a regular follow-up.

All the included patients underwent, unilateral frontotemporal or bifrontal approach according to the tumor size, location, extension and clinical features.

In case of midline tumors, a unilateral approach was generally performed on the side of the worse vision. We also recognized four fundamental maneuvers useful to unlock and mobilize the ON when necessary:

- FL section;
- optic canal unroofing, which provides a wider surgical corridors and early ON decompression and mobilization in vertical direction;
- anterior clinoid process drilling, sometimes until the optic strut exposure, in order to guarantee further ON mobilization in a medio-lateral direction;
- optic strut removal to obtain a circumferential optic nerve decompression and a lateral mobilization of the parasellar carotid artery.

All the decompressive procedures were intradural.

Extent of resection was assessed with Simpson score system at postoperative MRI.

Analysis of visual recovery was performed at 6 months.

Visual outcome was evaluated by considering changes in light perception in amaurotic patients, and objective measurements of visual acuity scores and visual field index testing results.

We analyzed the impact of various factors, including age, sex, duration of visual decline, direction of compression, duration of blindness, meningioma extension, extent of resection, and histopathology, on the visual outcome after surgical intervention. Statistical analysis was performed using SPSS software. Univariate analysis of clinical and surgical parameters was performed using Fisher’s exact test or Student’s T test/ANOVA as necessary. Stepwise binomial logistic regression was used to identify the best predictors of visual parameters’ improvement. Statistical significance was accepted at p values less than 0.05.

## Results

### Demographics

We included 40 patients, 7 males (17.5%) and 33 females (82.5%), with age ranging from 24 to 81 years (61.7 ± 12.4).

### Topographical and morphological characteristics of the tumors

**Fig. 1:**
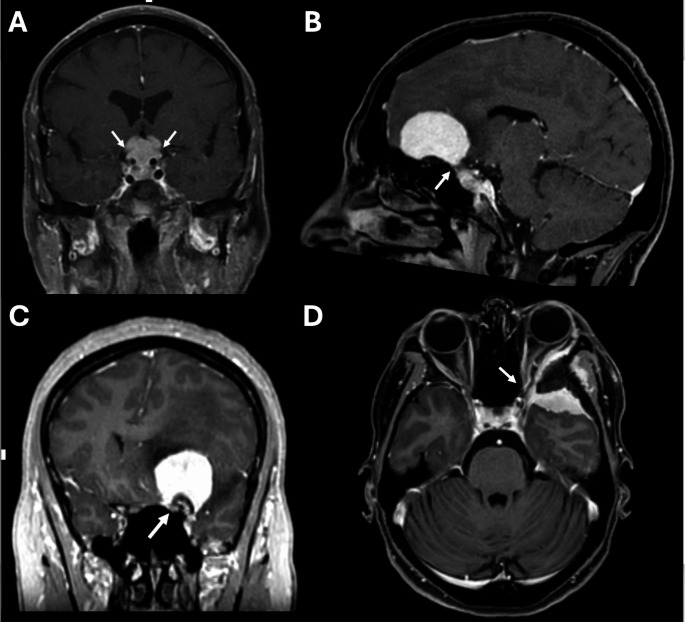
Panel showing the four types of anterior skull base meningiomas included in this study: meningiomas originating from tuberculum sellae with bilateral ONs compression (**A**); from sphenoid-ethmoidal planum with bilateral ONs compression (**B**); from anterior clinoidal process with unilateral ON compression (**C**), from sphenoid-orbital region with unilateral ON compression (**D**)

Meningiomas originated from anterior clinoidal process in 16 patients (40%), from sphenoid-ethmoidal planum in 11 (27.5%), from the tuberculum sellae in 10 (25%), from the sphenoid-orbital region in 3 (7.5% - Fig. 1).

Twenty-one patients had bilateral involvement of ONs (52.5%) due to tumors originating from tuberculum sellae in 10 cases, from sphenoid-ethmoidal planum in 10, and from anterior clinoidal process in 1.

In 19 patients (47.5%), instead, there was only an unilateral involvement of ON and the right side was the most frequently affected (13 right Vs 6 left side).

Four patients had encasement of the internal carotid artery (2 complete and 2 partial), 1 patient had encasement of the middle cerebral artery, and one had extension of the tumor into the cavernous sinus; a parasellar extension was observed in 15 out of 40 cases (37.6%).

Calcifications were present in 11/40 out of cases (24.3%) and preoperative hydrocephalus in 1 (2.5%).

According to the different vectors of compression, it emerged that a medial-to-lateral path was retrieved in 57.5%, a lateral-to-medial in 40% a superior-to-inferior 37.5%, an inferior-to-superior 15%, and finally, a posterior in 5%. Moreover, 15 patients had more than one path of compression at diagnosis (37.6%); in particular, the association between medial-to-lateral and cranial-to-caudal was the more frequent.

As regards to histopathology, 85% of the patients had a grade I meningioma, 12.5% a grade II and 2.5% a grade III according to WHO classification (Table 2).

**Table 2:** Radiological and histopathological characteristics of the tumors

Tumor size in mm, (mean ± sd)	37.5 ± 17.8
**Origin (epicenter)**	
Spheno-orbital	3 (7.5%)
Planum ethmoidalis, sphenoidalis	11 (27.5%)
Anterior Clinoid Process	16 (40.0%)
Tuberculum sellae	10 (25.0%)
**Direction of compression (vector)**	
Medial to lateral	23 (57.5%)
Lateral to medial	16 (40.0%)
Superior to inferior	15 (37.5%)
Inferior to superior	6 (15.0%)
Posterior	2 (5.0%)
**Histopathology (WHO 2021)**	
I	34 (85.0%)
II	5 (12.5%)
III	1 (2.5%)

### Clinical presentation

At admission, a visual examination documented a decline in visual acuity in 34/40 (85%) out of patients and in visual field in 28/40 (70%). Mean age appeared significantly lower in patients with intact visual field compared with those showing a narrowing (*p* = 0.006). Among them, a unilateral blindness was present in 9 cases (Table 3).

**Table 3:** Relationship between clinical variables and preoperative visual picture

Variable		Pre-Operative Visual Acuity		*p*	Pre-Operative Visual Field		*p*
		Deficit (*n* = 34)	Intact (*n* = 6)		Deficit (*n* = 28)	Intact (*n* = 12)	
Mean Age ± SD (years)		61.7 ± 12.8	61.2 ± 10.5	0.9	65 ± 10.7	53.8 ± 12.8	**0.006**
Female Sex (*n* = 33)		28 (82.4%)	5 (83.3%)	1	23 (82.1%)	10 (83.3%)	0.9
Mean Length of Visual Symptoms ± SD (months)		13.3 ± 17	7.2 ± 8.8	0.4	14 ± 18.1	8.6 ± 9.5	0.3
Mean Preoperative mRS ± SD		1.2 ± 0.5	1 ± 0.6	0.4	1.3 ± 0.5	0.9 ± 0.3	**0.03**
Mean Tumor Size ± SD (mm)		41 ± 14.9	38 ± 14.6	0.7	39.5 ± 14.4	42.5 ± 15.8	0.6
ON impingement	Bilateral (*n* = 18)	17 (50%)	1 (16.7%)	0.1	15 (53.6%)	3 (25%)	0.1
	Right only (*n* = 13)	12 (35.3%)	1 (16.7%)	0.4	9 (32.1%)	4 (33.3%)	0.9
	Left only (*n* = 9)	5 (14.7%)	4 (66.7%)	**0.005**	4 (14.3%)	5 (41.7%)	0.06
Tumor Origin	Spheno-Orbital (*n* = 3)	3 (8.8%)	0	0.4	3 (10.7%)	0	0.2
	Sphenoid Planum (*n* = 11)	9 (26.5%)	2 (33.3%)	0.7	8 (28.6%)	3 (25%)	0.8
	Anterior Clinoid (*n* = 16)	13 (38.2%)	3 (50%)	0.6	11 (39.3%)	5 (41.7%)	0.9
	Tuberculum Sellae (*n* = 10)	9 (26.5%)	1 (16.7%)	0.6	6 (21.4%)	4 (33.3%)	0.4
Vector of Compression	Medial to lateral (*n* = 23)	21 (61.8%)	2 (33.3%)	0.2	18 (64.3%)	5 (41.7%)	0.2
	Lateral to medial (*n* = 16)	13 (38.2%)	3 (50%)	0.6	10 (35.7%)	6 (50%)	0.4
	Superior to inferior (*n* = 15)	14 (41.2%)	1 (16.7%)	0.3	13 (46.4%)	2 (16.7%)	0.07
	Inferior to superior (*n* = 6)	4 (11.8%)	2 (33.3%)	0.2	2 (7.1%)	4 (33.3%)	**0.03**
	Posterior (*n* = 2)	1 (2.9%)	1 (16.7%)	0.2	1 (3.6%)	1 (8.3%)	0.5
Visual Deficit Outcome	Improved	17 (50%)	-	-	8 (28.6%)	-	-
	Stable	14 (41.2%)	6 (100%)	**0.008**	15 (53.6%)	12 (100%)	**0.004**
	Worsened	3 (8.8%)	0	0.4	5 (17.6%)	0	0.1

Five patients (15%) presented a clinical onset with other symptoms such as anosmia, abulia and hypothalamic disturbances, while headache was in general the third most common symptom (7.5%). An incidental finding was reported in 5% out of cases. The mean lasting of symptoms was 12.4 ± 16.1 months (Table 4).

**Table 4:** Preoperative patient’s clinical characteristics

**Clinical onset**	
Ipovisus	34 (85%)
Deficit visual field	28 (70%)
Seizure	1 (2.5%)
Headache	3 (7.5%)
Other	2 (5%)
Incidental	2 (5%)
**Lasting of symptoms** (month), mean ± sd	12.4 ± 16.1

VF = Visual Field

Among patients with impaired visual acuity, 17 (50%) had a bilateral ON impingement, 12 (35.3) a unilateral right contact, and 5 (14.7%) a unilateral left. In the last group, we observed a significant prevalence of asymptomatic patients. Similar percentages were observed among patients according to the pre-operative visual field impairment (Table 3).

No differences were instead observed between symptomatic and asymptomatic patients according to the tumor origin, whereas a significantly lower rate of visual field cut was observed among patients with an inferior-to-superior compression. On the contrary, tumors determining superior-to-inferior compression showed a trend with higher risk of visual field sufferance (Table 3).

### Surgery and complications

The majority of patients underwent a right pterional approach (60%); a left pterional was instead used in 22.5% of cases, and a bifrontal in 17.5%.

**Fig. 2:**
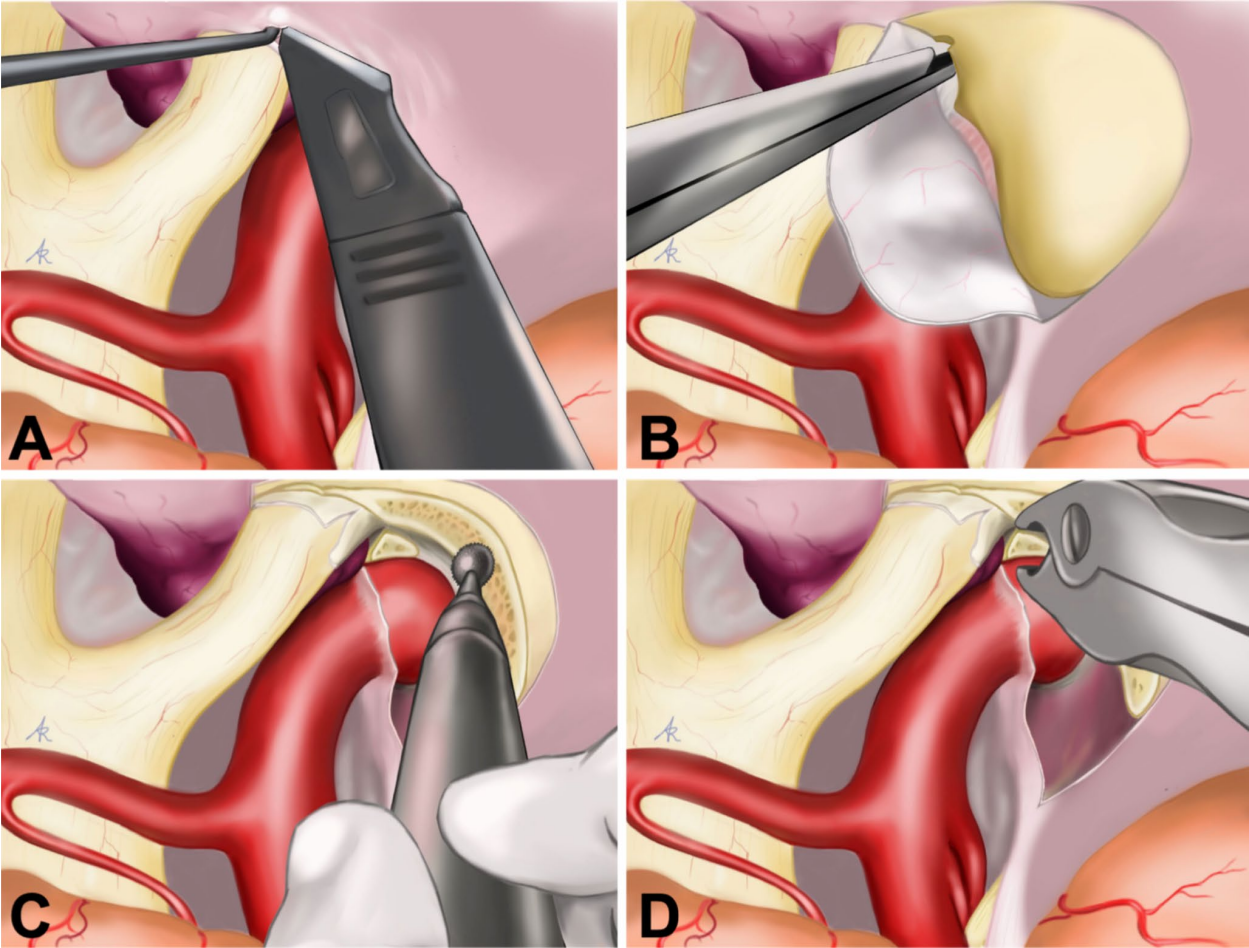
Original artwork showing the 4 most important maneuvers for ON unlocking: microsurgical dissection of the basal cisterns and FL opening (panel **A**); unroofing of the ON canal (panel **B**); anterior clinoidectomy (panel **C**); optic strut removal and ON mobilization (panel **D**)

**Fig. 3:**
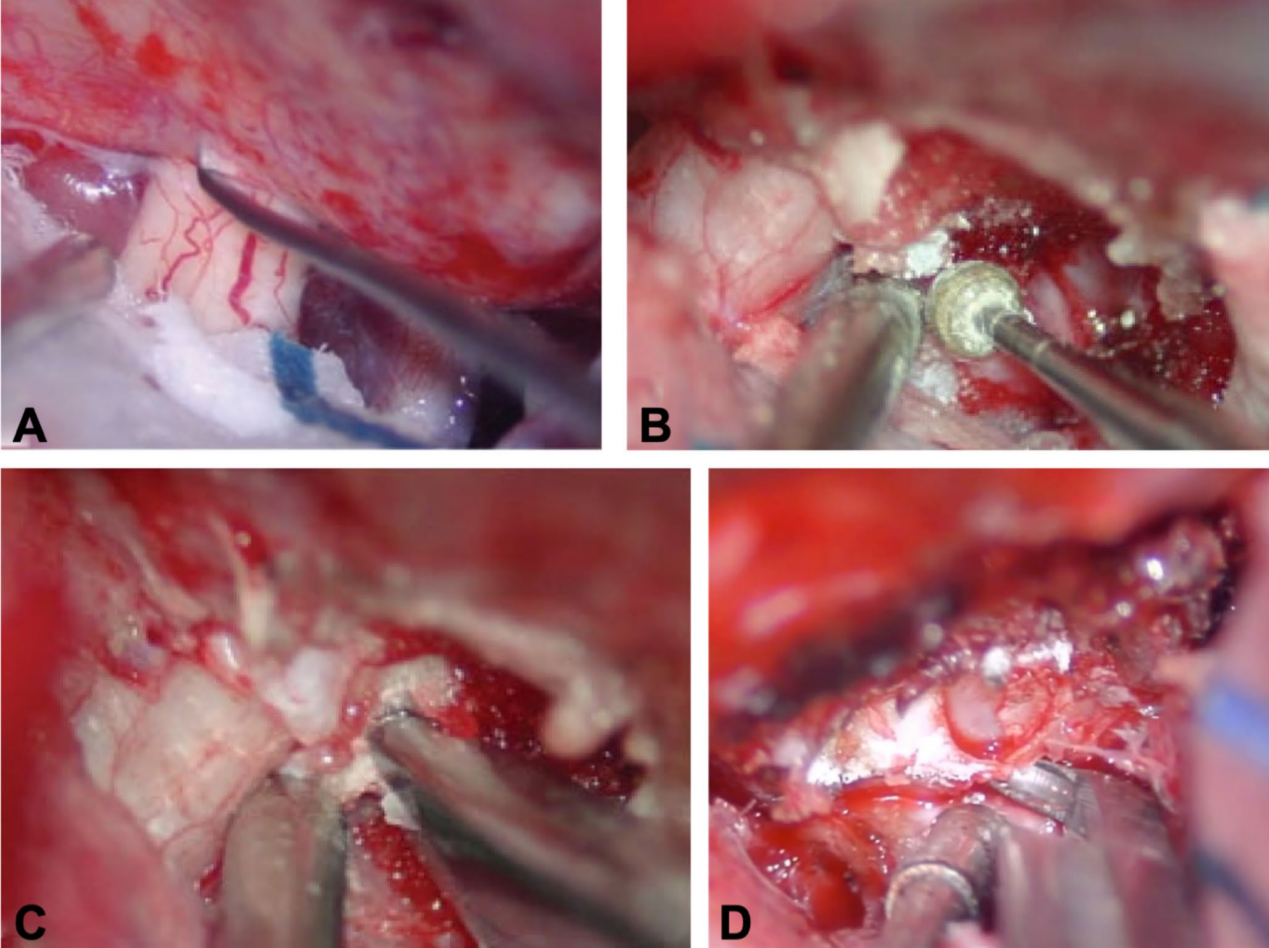
Intraoperative views showing the identification of falciform ligament and its immediate cutting before tumor manipulation (**A**); the drilling of the anterior clinoid process until the exposure of the optic strut (**B**); optic strut removal with a Kerrison rongeur (**C**); the optic canal unroofing and nerve decompression (**D**)

All patients underwent the necessary maneuvers of ON unlocking before its direct or indirect mobilization during the tumor debulking (Table 1; Figs. 2 and 3). The opening of the FL was performed in most of patients (82.5%), associated with unroofing of optic canal in 27%, anterior clinoidectomy in 32%, and opening of optic strut in 5%. At surgery, a macroscopically complete removal (Simpson grade 1–2) was obtained in almost ¾ of patients (72.5%) and post-operative MRI confirmed a gross total resection in 77.5% out of patients (Table 5).

**Table 5:** Surgical approach and Simpson grading

**Type of approach**	
Right pterional	24 (60%)
Left pterional	9 (22.5%)
Bifrontal	7 (17.5%)
**Optic nerve unlocking technique**	
Falciform ligament (FL)	33 (82.5%)
FL + Unroofing	11 (27.5%)
FL + Clinoidectomy	13 (32%)
FL + Optic Strut	2 (5%)
**Simpson grade**	
1	0 (0%)
2	29 (72.5%)
3	2 (5%)
4	9 (22.5%)
5	0 (0%)

FL = Falciform Ligament

No recurrences were observed among patients with gross total resection, whereas 4/9 out of patients with subtotal removal showed progression of the residual over time and underwent fractionated radiotherapy, with a tumor control at follow up.

Complications were observed in 4/40 out of patients (10%): 1 showed hemiparesis in 1 patient, 1 right frontal lobe ischemia and 2 hemorrhagic infarctions of the surgical bed.

The first two cases showed a complete encasement of internal carotid artery and his major branches, whereas the second two cases had in common larger size of the tumor.

### Post-operative visual outcome

Visual outcome was assessed at a 6-month follow-up.

Among patients treated before the onset of visual acuity disturbances, none experienced post-operative worsening (Table 3). Instead, among those with preoperative deficits, 50% improved, 41.2% remained unchanged, and 8.8% worsened.

The only significant predictor of worsening was the mean duration of symptoms (46 ± 34 months) between stable (9.17 ± 8.2 months) and improved (10.17 ± 13.29 months) patients (*p* < 0.001). No significant differences in the direction of optic pathway compression were observed among these outcome groups.

Due to the low rate of worsened patients, we focused our analysis on factors associated with non-improvement. At univariate analysis, only the optic canal unroofing appeared significantly associated with non-improvement.

A stepwise binomial logistic regression analysis, excluding asymptomatic preoperative patients, identified a statistically significant model (*p* = 0.03; AUC = 0.796) with optic canal unroofing as the only significant predictor of postoperative non-improvement (OR = 0.163; 95%CI: 0.027–0.983; *p* = 0.04).

Similar results were observed according to visual field cut: those without preoperative deficit did not developed visual field cut after surgery. On the contrary only 28.6% of patients with preoperative deficits experienced post-operative improvement and a worsening occurred in 17.6% of those with preoperative visual field deficits; the remaining 53.6% showing no change at 6-month. Interestingly, worsened cases had longer durations of preoperative symptoms compared to those who remained unchanged or improved (*p* = 0.04).

Comparing patients with and without post-operative visual field improvement, only younger age and better preoperative mRS status were significantly associated with positive outcome. A stepwise binomial logistic regression model, excluding asymptomatic preoperative patients, identified age as a significant risk factor for lacking of post-operative improvement (OR = 0.855; 95%CI: 0.743–0.983; *p* = 0.028). This suggests that older patients may be less likely to experience positive outcomes following the procedure (Tables 6 and 7).

**Table 6:** Comparison between patients with e without post-operative visual acuity improvement

Variable		Post-Operative Visual Acuity (*n* = 34)		*p*
		Improved (*n* = 17)	Not-Improved (*n* = 17)	
Mean Age ± SD (years)		59.9 ± 15.25	63.5 ± 9.9	0.4
Female Sex (*n* = 28)		15 (88.2%)	13 (76.5%)	0.4
Mean Length of Visual Symptoms ± SD (months)		10.2 ± 13.3	16.4 ± 20	0.3
Mean Preoperative mRS ± SD		1.06 ± 0.43	1.35 ± 0.49	0.07
Mean Tumor Size ± SD (mm)		44.44 ± 14.9	37.27 ± 14.42	0.2
Preoperative Blindness or Severe VA Deficit (*n* = 19)		10 (58.9%)	9 (53%)	0.7
Preoperative VF Deficit (*n* = 27)		14 (82.35%)	13 (76.5%)	0.7
Tumor Origin	Spheno-Orbital (*n* = 3)	0	3 (17.65%)	0.07
	Sphenoid Planum (*n* = 9)	6 (35.3%)	3 (17.65%)	0.2
	Anterior Clinoid (*n* = 13)	6 (35.3%)	7 (41.2%)	0.7
	Tuberculum Sellae (*n* = 9)	5 (29.4%)	4 (23.5%)	0.7
Vector of Compression	Medial to lateral (*n* = 21)	12 (70.6%)	9 (53%)	0.3
	Lateral to medial (*n* = 13)	5 (29.4%)	8 (47%)	0.3
	Superior to inferior (*n* = 14)	6 (35.3%)	8 (47%)	0.5
	Inferior to superior (*n* = 4)	1 (5.9%)	3 (17.65%)	0.3
	Posterior (*n* = 1)	1 (5.9%)	0	0.3
Surgical Approach	Right Craniotomy (*n* = 21)	8 (47%)	13 (76.5%)	0.08
	Left Craniotomy (*n* = 7)	4 (23.5%)	3 (17.65%)	0.7
	Bilateral Craniotomy (*n* = 6)	5 (29.4%)	1 (5.9%)	0.07
Optic Nerve Unlocking Maneuver	Falciform Ligament Opening (*n* = 30)	14 (82.35%)	16 (94.1%)	0.3
	Optic Canal Unroofing (*n* = 11)	2 (11.8%)	9 (53%)	**0.01**
	Anterior Clinoidectomy (*n* = 4)	1 (5.9%)	3 (17.65%)	0.3
	Optic Strut Removal (*n* = 2)	0	2 (11.8%)	0.1
Tumor Grade	WHO Grade 1 (*n* = 28)	16 (94.1%)	12 (70.6%)	0.07
	WHO Grade 2 (*n* = 5)	1 (5.9%)	4 (23.5%)	0.15
	WHO Grade 3 (*n* = 1)	0	1 (5.9%)	0.3
Extent of resection	Simpson Grade 1 (*n* = 0)	0	0	-
	Simpson Grade 2 (*n* = 25)	13 (76.5%)	12 (71%)	0.7
	Simpson Grade 3 (*n* = 2)	1 (5.9%)	1 (5.9%)	1
	Simpson Grade 4 (*n* = 7)	3 (17.65%)	4 (23.5%)	0.7
	Simpson Grade 5 (*n* = 0)	0	0	-

**Table 7:** Comparison between patients with e without post-operative visual field improvement

Variable		Post-Operative Visual Field (*n* = 28)		*p*
		Improved (*n* = 8)	Not-Improved (*n* = 20)	
Mean Age ± SD (years)		57 ± 11.2	68 ± 9	**0.01**
Female Sex (*n* = 28)		7 (87.5%)	16 (80%)	0.6
Mean Length of Visual Symptoms ± SD (months)		16.5 ± 15.8	13 ± 19.3	0.6
Mean Preoperative mRS ± SD		0.87 ± 3.5	1.45 ± 0.5	**0.007**
Mean Tumor Size ± SD (mm)		42.87 ± 14.35	34.94 ± 14.52	0.4
Preoperative Blindness or Severe VA Deficit (*n* = 19)		6 (75%)	10 (50%)	0.2
Tumor Origin	Spheno-Orbital (*n* = 3)	0	3 (15%)	0.2
	Sphenoid Planum (*n* = 8)	3 (37.5%)	5 (25%)	0.5
	Anterior Clinoid (*n* = 11)	2 (25%)	9 (45%)	0.3
	Tuberculum Sellae (*n* = 6)	3 (37.5%)	3 (15%)	0.2
Vector of Compression	Medial to lateral (*n* = 18)	7 (87.5%)	11 (55%)	0.1
	Lateral to medial (*n* = 10)	1 (12.5%)	9 (45%)	0.1
	Superior to inferior (*n* = 2)	2 (25%)	11 (55%)	0.15
	Inferior to superior (*n* = 4)	1 (12.5%)	1 (5%)	0.5
	Posterior (*n* = 1)	0	1 (5%)	0.5
Surgical Approach	Right Craniotomy (*n* = 16)	4 (50%)	12 (60%)	0.6
	Left Craniotomy (*n* = 7)	2 (25%)	5 (25%)	1
	Bilateral Craniotomy (*n* = 5)	2 (25%)	3 (15%)	0.5
Optic Nerve Unlocking Maneuver	Falciform Ligament Opening (*n* = 25)	7 (87.5%)	18 (90%)	0.85
	Optic Canal Unroofing (*n* = 9)	2 (25%)	7 (35%)	0.6
	Anterior Clinoidectomy (*n* = 5)	1 (12.5%)	4 (20%)	0.6
	Optic Strut Removal (*n* = 2)	0	2 (10%)	0.3
Tumor Grade	WHO Grade 1 (*n* = 25)	8 (100%)	17 (85%)	0.25
	WHO Grade 2 (*n* = 5)	0	3 (15%)	0.25
	WHO Grade 3 (*n* = 0)	0	0	-
Extent of resection	Simpson Grade 1 (*n* = 0)	0	0	-
	Simpson Grade 2 (*n* = 21)	6 (75%)	15 (75%)	1
	Simpson Grade 3 (*n* = 1)	0	1 (5%)	0.5
	Simpson Grade 4 (*n* = 6)	2 (25%)	4 (20%)	0.8
	Simpson Grade 5 (*n* = 0)	0	0	-

## Discussion

The surgical management of anterior cranial fossa meningiomas associated with ONs compression presents unique challenges due to the relationship between the tumor and critical neurovascular structures. The primary goals of surgery is ONs decompression and mass effect reduction. Although a complete tumor resection is desirable, it should never be prioritized over the preservation of visual function. Nowadays, in fact, alternative strategies for managing anterior cranial fossa meningiomas include observation and radiation therapy in case of tumor growing, which can effectively manage residual tumor without compromising vision. However, the common purpose of all three approaches is to stabilize or hopefully improve the vision of the ipsilateral eye. On the other hand, for patients with total blindness of the ipsilateral eye, the primary goal is to preserve vision in the contralateral eye [3, 5, 16].

Surgical outcomes for anterior cranial fossa meningiomas vary depending on the topography of ON compression. A recent systematic review by Henaux et al. found that patients with isolated cisternal ON compression had higher rates of postoperative visual improvement compared to those with optic canal involvement. Moreover, patients with intraorbital meningiomas had the worst outcomes, with low rates of visual improvement and high rates of visual worsening [5].

Transcranial routes have been traditionally preferred by the majority of neurosurgeons for ONs decompression, although minimally invasive endonasal and trans-orbital approaches have gained popularity in recent decades.

To preserve the integrity of the optic pathways, the surgical strategy should be tailored according to the principle of “unlock the nerve structures at first” allowing for greater surgical freedom of ON during tumor manipulation. This can be obtained through sectioning or drilling osteo-dural structures that anatomically constrain the ONs.

Unlocking the ON may require a series of sequential procedures such as FL section, optic canal unroofing, drilling of the anterior clinoid process, and removal of the optic strut according to the desired degree of freedom of ON. The choice of technique depends on the tumor’s size, topography and direction of ON compression.

Optic nerve decompression and the other releasing maneuvers should be in general performed as a first stage of surgery based on the anatomy and topography of the meningioma and virtually before any manipulation of the tumor that may transmit the traction to optic nerve or chiasm. For larger meningiomas, the necessary unlocking maneuvers often need to be preceded by a partial lesion debulking. In case of optic canal involvement, after the debulking of a significant amount of tumor and the exposure of the cisternal course of the ON, section of FL and unroofing of the optic canal takes place. Then, dura over optic canal is incised and optic roof is drilled using a 3-mm diamond drill, abundantly irrigating to minimize the risk of thermal injury of optic nerve. Before to start drilling, it is always mandatory to remove all cottonoids to avoid entanglement of the rotating ball. When a thin plate of bone is left on the canal roof, it can be easily removed with a dissector until exposing the lateral walls of the optic canal. Then, the residual tumor around the nerve can be progressively removed until to achieve a gross total resection (GTR) by identifying the arachnoid plane preserving the ON and its vascular supply and avoiding undesired tractions.

Another important anatomical aspect that should be taken into account during the surgical planning is the relationship between the meningioma and the vascular structures, in particular the internal carotid artery and its branches, including perforating and ophthalmic branch. These relationships can be studied with non-invasive neuroimaging techniques or when necessary with preoperative DSA, since the importance of vascular sparing is equal, if not greater, to that of the nervous structures.

The involvement of the optic pathways is frequent in certain anterior cranial fossa meningiomas that may clinically present visual deterioration. In our 5-year retrospective study, we identified 40 patients harboring anterior cranial fossa meningiomas with radiological impingement of one or both ONs. We found that the most important factor influencing post-operative visual outcome was the presence of pre-operative visual deficits. Patients who underwent surgery before the onset of symptoms showed an intact vision after surgery in all cases supporting our strategy to unlock ON as a first step. On the other hand, only a portion of patients with preoperative deficits experienced improvement. Moreover, we also observed that the pre-operative symptoms duration was longer in those few patients who showed a post-operative worsening in visual functions.

Age seems play a role since younger patients have a lower rate of preoperative visual field cut despite similar optic pathways compression of older people and therefore an early intervention may provide a higher chance of a better visual outcome. Multivariate analysis in fact confirmed that older patients less probably experience positive outcomes following decompressive procedures.

These findings emphasize the importance of early intervention, especially for patients with recent symptoms onset and tumors compressing the ONs with superior-to-inferior direction. While tumor size did not seem directly correlated with visual outcome, tumor location and direction of ONs appeared crucial factors. The absence of a strict relationship between tumor size and symptoms in our series, can be explained by the fact that we collected meningiomas of the anterior skull base with different origins, which may determine optic pathways impingement with different timing during their natural history due to the distance between the side of origin and ONs course.

We also compared patients showing visual improvement with those who not according to demographics and morphological characteristics of the tumor, vector of compression, surgical technique and amount of tumor resection, and observed that only those who underwent optic canal unroofing showed a significantly higher rate of non-improvement in visual acuity after ON decompression. This is probably attributable to the fact that they had meningiomas invading the optic canal with a more severe ON compression. The extension into the optic canal therefore represents the most severe evolution of these anterior skull base meningiomas, since in these cases, despite the preliminary nerve unlocking maneuvers and the careful microsurgical ON decompression, the probability of recovery of visual acuity remains low.

Moreover, some other anatomical variations of the optic apparatus can influence the risk of visual impairment. A pre-fixed chiasm, for example, may predispose to early visual deficits, while a post-fixed chiasm may delay diagnosis. Additionally, the presence of a tough environment in the intracranial space or orbit can hinder early detection of ON compression [4].

Not only the location of ON compression but also the ON environment significantly influence diagnosis delay. Absence of tough environment in IC space and orbit can lead to delayed diagnosis of visual impairment. Hence, the ON meningiomas most liable to be detected early via visual impairment are those located in the optic canal [10, 14].

Finally, the relationship between the tumor and ON is another important factor. Some meningiomas may push the ON, but preserve the arachnoid plane, while others may encase the ON or grow entirely within its sheath. These different growing pathways can affect the timing of visual deficit appearance and influence the surgical management and outcome [1, 9, 11].

## Conclusions

The surgical management of anterior cranial fossa meningiomas associated with optic nerve compression should prioritize visual preservation over radical tumor resection. Early intervention, meticulous preoperative planning, and precise surgical techniques are crucial for optimizing outcomes as a timely decompression seems to reduce the risk of post-operative visual acuity deterioration, whereas a late decompression shows limited opportunity of visual improvement. To minimize ON manipulation and maximize the likelihood of preserving visual function, surgical techniques should be modified to include all the necessary unlocking strategies that reduce stress on the optic nerve during the tumor resection.

## Data Availability

No datasets were generated or analysed during the current study.

## Abbreviations

ONs: Optic nerves
FL: Falciform ligament
